# 53BP1: a DSB escort

**DOI:** 10.1101/gad.333237.119

**Published:** 2020-01-01

**Authors:** Zachary Mirman, Titia de Lange

**Affiliations:** Laboratory for Cell Biology and Genetics, The Rockefeller University, New York, New York 10065, USA

**Keywords:** 53BP1, BRCA1, CSR, CST, DNA, PARP1, PARPi, Shieldin, telomere, double-strand break

## Abstract

53BP1 is an enigmatic DNA damage response factor that gained prominence because it determines the efficacy of PARP1 inhibitory drugs (PARPi) in BRCA1-deficient cancers. Recent studies have elevated 53BP1 from its modest status of (yet another) DNA damage factor to master regulator of double-strand break (DSB) repair pathway choice. Our review of the literature suggests an alternative view. We propose that 53BP1 has evolved to avoid mutagenic repair outcomes and does so by controlling the processing of DNA ends and the dynamics of DSBs. The consequences of 53BP1 deficiency, such as diminished PARPi efficacy in BRCA1-deficient cells and altered repair of damaged telomeres, can be explained from this viewpoint. We further propose that some of the fidelity functions of 53BP1 coevolved with class switch recombination (CSR) in the immune system. We speculate that, rather than being deterministic in DSB repair pathway choice, 53BP1 functions as a DSB escort that guards against illegitimate and potentially tumorigenic recombination.

## Abstract (precis)

In this review, Mirman et al. summarize the current understanding of the role of 53BP1 in DSB repair at deprotected telomeres, in class switch recombination in the immune system, and in the context of PARPi-treated BRCA1-deficient cells. They argue that the primary function of 53BP1 is not to regulate the choice between c-NHEJ and HDR, but to ensure the fidelity of DSB repair, a function that is corrupted in diseases where DNA repair is rewired.

Escort: late 16th century (originally denoting a body of armed men escorting travelers): from French escorte (noun), escorter (verb); from Italian scorta, feminine past participle of scorgere “to conduct, guide,” based on Latin ex- “out of” + corrigere “set right”

—Oxford Dictionary of English

Every eukaryotic cell contends with a staggering variety and quantity of threats to its DNA, with insults generating double-strand breaks (DSBs) representing perhaps the most toxic events. A crucial, yet enigmatic player in the repair of DSBs is 53BP1 (p53-binding protein 1, also known as TP53BP1). Although 53BP1 was discovered and named based on its interaction with p53 (Iwabuchi et al. 1994), 53BP1 has been most thoroughly characterized in terms of its role at broken DNA ends where it recruits effector proteins to mediate DSB repair (Panier and Boulton 2014; Zimmermann and de Lange 2014). Chief among these effector proteins are those that affect the formation of single-strand DNA (ssDNA) at breaks, which is an important step in repair of physiological and pathological DSBs (Symington and Gautier 2011; Hustedt and Durocher 2017).

DSBs are repaired by two distinct pathways: classical nonhomologous end-joining (c-NHEJ) and homology-directed repair (HDR) (Fig. 1). c-NHEJ ligates blunt DNA ends or DSBs with short overhangs (Fig. 1A; Pannunzio et al. 2018). This pathway is initiated by DNA end-binding of the ring-shaped Ku70/80 heterodimer, which also can hold two DNA ends together (Soutoglou et al. 2007). Ligation of the ends requires DNA Ligase IV (Lig4) together with its associated factors (e.g., XRCC4 and XLF). This process requires the catalytic subunit of DNA protein kinase (DNA-PKcs) and, in some settings, is promoted by nucleases (e.g., Artemis) and polynucleotide kinase 3′ phosphatase (PNKP). c-NHEJ is rapid, efficient, and very accurate when the DNA ends are “clean” (have compatible or blunt ends not blocked by attached proteins). Although active throughout interphase, c-NHEJ can be inhibited in S/G2 by CYREN (also called MRI) (Agarwal et al. 2006; Grundy et al. 2016) if DSBs contain 5′ or 3′ overhangs (Arnoult et al. 2017). In contrast, CYREN/MRI stimulates c-NHEJ in G1 (Hung et al. 2018).

**Figure 1.**
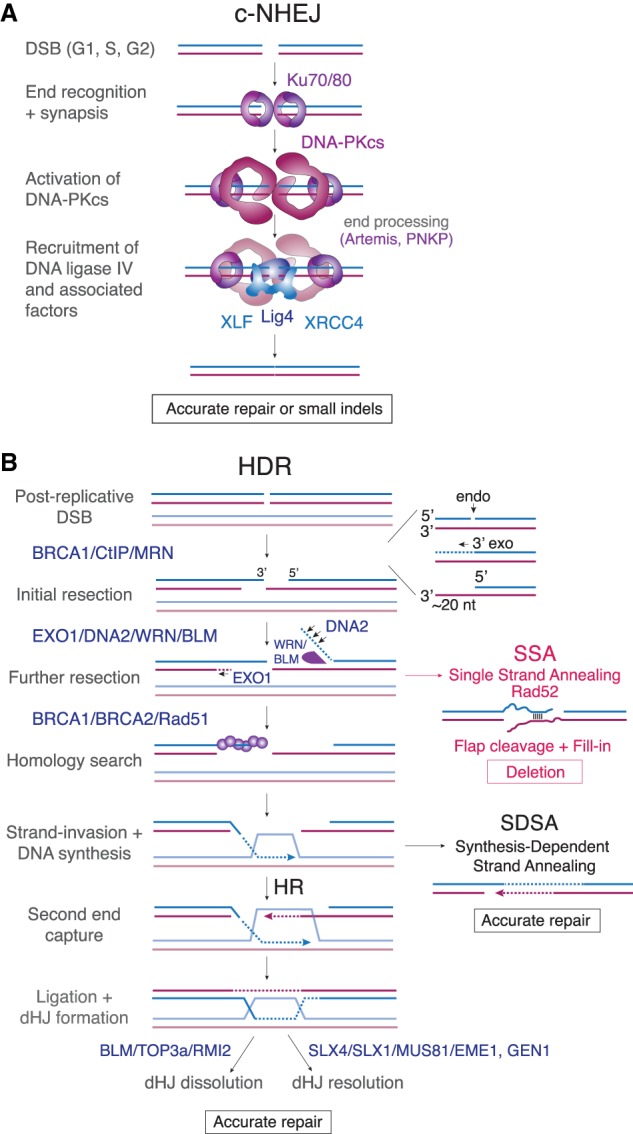
The two predominant DSB repair pathways in mammalian cells. (*A*) Schematic of c-NHEJ repair of blunt or minimally processed DNA ends. This pathway is active throughout the cell cycle and the result is accurate repair or small insertions or deletions (indels). (*B*) Schematic of HDR of postreplicative (S/G2) DSBs. This pathway requires the generation of 3′ overhangs competent for loading Rad51. Single-strand annealing (SSA), resulting in deletions, can also occur if excessive resection exposes regions of homology. Accurate repair is achieved either by SDSA or HR followed by dissolution or resolution of the dHJ.

Unlike c-NHEJ, HDR of DSBs (Fig. 1B) requires the presence of a 3′ overhang (Wright et al. 2018). When coated with the Rad51 recombinase, 3′ overhangs can initiate the critical strand-invasion event required for HDR. Accurate HDR uses the sister chromatid as a template and DSB repair by HDR is therefore prominent in newly replicated parts of the genome. The critical step toward HDR, formation of the 3′ overhang, is thought to require BRCA1 (Roy et al. 2011). Resection of the 5′ end is initiated by the MRN (MRE11, RAD50, and NBS1) complex in conjunction with CtIP (Sartori et al. 2007). MRN/CtIP binds to DSBs and possesses both endonucleolytic and 3′ exonucleolytic activity allowing formation of a short oligonucleotide that can be degraded from its 3′ end (Garcia et al. 2011; Anand et al. 2016, 2019; Deshpande et al. 2016; Myler et al. 2017). This process creates a short 3′ overhang that can be further processed by long-range 5′ end resection either by the processive exonuclease EXO1 (Garcia et al. 2011), or by the flap endonuclease DNA2 acting on ssDNA formed by BLM or WRN helicase activity (Nimonkar et al. 2011). BRCA2-mediated loading of Rad51 on the ssDNA creates the Rad51 nucleofilament that executes a homology search (Davies et al. 2001; Moynahan et al. 2001). The resulting strand invasion generates the substrate for DNA synthesis, which elongates the 3′ overhang in a templated fashion. Capture and ligation of the other DNA end generates a double Holliday junction (dHJ) that can be dissolved through migration by the BTR (BLM–TOP3A–RMI) complex to separate the sister chromatids without a crossover. Alternatively, persistent dHJs can be resolved later in the cell cycle by HJ resolvases such as the SLX4/SLX1/Mus81/EME1 complex, and GEN1, yielding separated sister chromatids with or without a crossover (West et al. 2015). If the second end is not captured, the extended overhang can anneal to sequences at the second end (after resection) in a process called synthesis-dependent strand annealing (SDSA) (Fig. 1B). SDSA leads to accurate repair of DSBs without crossovers.

Although 3′ overhang formation is critical for HDR, excessively long 3′ overhangs at DSBs can be dangerous if they contain repetitive sequences that allow the Rad52-dependent single-strand annealing (SSA) pathway to create deletions (Fig. 1B). DNA breaks with 3′ overhangs can also be processed by a second mutagenic DNA repair pathway, referred to as alternative NHEJ (alt-NHEJ) or microhomology-mediated end joining (MMEJ). In mammalian cells, alt-NHEJ requires only minimal homology (one or more base pairs) between regions of ssDNA and is mediated by enzymes normally involved in base excision repair (BER), such as the poly(ADP-ribose) polymerase PARP1 and Ligase 3, as well as the error-prone PolQ polymerase (Sfeir and Symington 2015).

Because of the dependence of accurate HDR (homologous recombination or SDSA) on single-stranded overhangs, the structure of the DSB is a determinant of the repair pathway choice. It is at this node that 53BP1 has been implicated. Experiments involving dysfunctional telomeres, CSR, and PARPi-treated BRCA1-deficient cells established that a central function of 53BP1 at sites of DNA damage is to limit the formation of long 3′ protrusions (Bouwman et al. 2010; Bunting et al. 2010; Di Virgilio et al. 2013; Zimmermann et al. 2013). The mechanism by which 53BP1 controls the structure at DNA ends is still elusive. In addition to regulating DNA end processing, 53BP1 has the ability to endow DSBs with greater mobility in the nucleus and can promote synapsis of DNA breaks (Difilippantonio et al. 2008; Lottersberger et al. 2015).

The finding that 53BP1 deficiency leads to diminished c-NHEJ in the settings discussed above has resulted in the proposal that this factor is critical for c-NHEJ. However, unlike core c-NHEJ factors, loss of 53BP1 confers only mild sensitivity to DNA damage caused by ionizing radiation (IR) (Morales et al. 2003) or chemical insults (Xu et al. 2017). Similarly, although 53BP1 deficiency slightly reduces and delays the c-NHEJ of a subset of “normal” DSBs (e.g., those induced by IR) (Noon et al. 2010), this effect is minor compared with the loss of core c-NHEJ factors (Xu et al. 2017). Furthermore, unlike the core c-NHEJ factors, 53BP1 is not required for c-NHEJ of most RAG-induced DNA breaks during V(D)J recombination (Ward et al. 2004). Clearly, 53BP1 is not a core c-NHEJ factor but can increase the use of c-NHEJ in certain settings.

This review summarizes the current understanding of the role of 53BP1 in DSB repair at deprotected telomeres, in CSR, and in the context of PARPi-treated BRCA1-deficient cells. We argue that the primary function of 53BP1 is not to regulate the choice between c-NHEJ and HDR, but to ensure the fidelity of DSB repair, a function that is corrupted in diseases where DNA repair is rewired, as in BRCA1-deficient cancers.

## 53BP1

**Figure 2.**
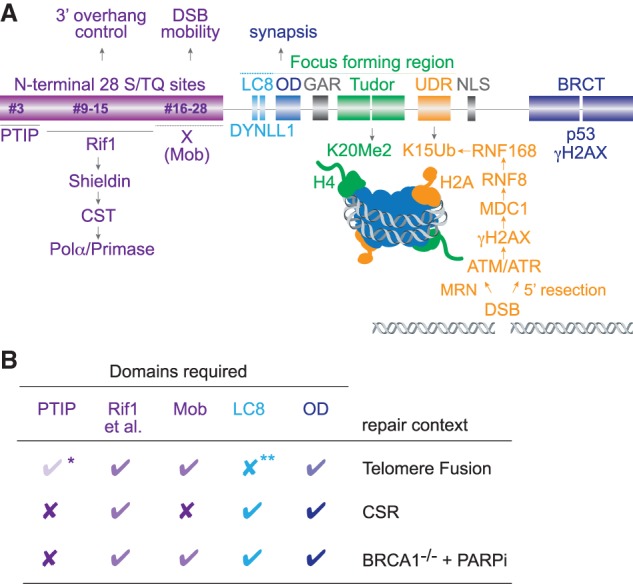
Domain structure and function of 53BP1. (*A*) Schematic of human 53BP1. Recruitment of 53BP1 to DSBs requires the Focus Forming Region (FFR), comprising the oligomerization domain (OD), the glycine–arginine-rich (GAR) motif, the tandem Tudor domain, the ubiquitin-dependent recognition (UDR) motif, and the dynein light chain (LC8) binding domain. The N-terminal S-T/Q phosphorylation sites mediate interactions with PTIP, the RIF1/Shieldin/CST/Polα/Primase axis, and an as-yet-unidentified factor (X) that promotes DSB mobility (Mob). The S-T/Q site numbers refer to #3 (S25), #9–15 (T302, S437, S452, S523, S543, S580, S625), and #16–28 (S674, T696, S698, S784, S831, T855, S892, S1068, S1086, S1104, S1148, T1171, S1219) (Bothmer et al. 2011). (*B*) Effects of absence of interactions between 53BP1 domains and the indicated interacting partners on c-NHEJ in three contexts. X indicates no requirement; a check mark indicates a requirement. Darker check marks indicate greater dependency. Nuclear localization of 53BP1 and its ability to form foci are required in all three settings; the BRCT domains are not required for any of the three c-NHEJ reactions. (*) There are conflicting results on the role of PTIP in c-NHEJ of TRF2-deficient telomeres with one study showing a strong effect (Callen et al. 2013) and two studies showing no (or a minor) effect (Lottersberger et al. 2013; Boersma et al. 2015); (**) the requirement for LC8 in telomere fusions has not been tested in the context of full-length 53BP1.

53BP1 is a large scaffold protein composed of domains that mediate interactions with modified histones and several effector proteins (Fig. 2A; Panier and Durocher 2013; Panier and Boulton 2014). The best-understood region of the 53BP1 protein is the central focus forming region (FFR), which is the minimal region required for accumulation of 53BP1 in chromatin near DSBs. It spans the oligomerization domain (OD), a glycine/arginine-rich (GAR) motif, a tandem Tudor domain, and a ubiquitin-dependent recruitment (UDR) motif (Fig. 2A).

The FFR interacts with modified histones in a manner dependent on the DNA damage response (DDR). At DSBs, DNA damage signaling is initiated by association of MRN with DNA ends and subsequent activation of MRN-bound ataxia telangiectasia mutated (ATM) kinase. ATM signaling initiates a cascade of chromatin phosphorylation and ubiquitination events, leading to recognition of chromatin by the 53BP1 FFR. Phosphorylation of histone H2AX (at S139, termed γH2AX) is followed by recruitment of MDC1 (Lou et al. 2003; Shang et al. 2003). Next, two E3 ubiquitin ligases, RNF8 and RNF168, ubiquitinate targets in chromatin surrounding the DSB, including H2AK15 (Huen et al. 2007; Kolas et al. 2007; Mailand et al. 2007; Doil et al. 2009; Stewart et al. 2009; Mattiroli et al. 2012). The 53BP1 FFR recognizes H2AK15-Ub with its UDR motif (Fradet-Turcotte et al. 2013), while its tandem Tudor domain binds H4K20me2—a mark present throughout the genome (Fig. 2A; Botuyan et al. 2006). In the absence of DNA damage, the Tudor domain of 53BP1 can associate with TIRR (Tudor-interacting repair regulator; also known as Syndesmos or Nudt16L1), which diminishes the interaction of 53BP1 with H4K20me2 (Drané et al. 2017; Zhang et al. 2017; Botuyan et al. 2018; Dai et al. 2018). TIRR binding is disrupted by DNA damage signaling, improving the ability of 53BP1 to associate with H4K20me2. Thus, upon activation of the DDR two histone marks, H4K20me2 and H2AK15Ub, engage the FFR and lead to 53BP1 accumulation at chromatin proximal to DSBs.

The accumulation of 53BP1 at sites of DNA damage is further stimulated by two domains: the oligomerization domain (OD) and the LC8 domain (Fig. 2A). The OD mediates homotypic interactions forming 53BP1 dimers and multimers independent of DNA damage signaling (Adams et al. 2005). This domain promotes 53BP1 recruitment to IR-induced DSBs (Ward et al. 2006; Zgheib et al. 2009), DSBs formed during CSR (Bothmer et al. 2011), and has a (minor) effect on the localization of 53BP1 to damaged telomeres (Fig. 2B; Lottersberger et al. 2013). The OD also promotes self-assembly of 53BP1 into phase-separated condensates (Kilic et al. 2019). This feature could explain the 53BP1-mediated synapsis of distal DSBs, which occurs in certain contexts [e.g., V(D)J recombination of distal RAG sites and dysfunctional telomeres] (see below). Binding of the LC8 domain to dynein light chain (DYNLL1) can also promote 53BP1 oligomerization and stimulate the recruitment of 53BP1 to sites of DNA damage (Becker et al. 2018; He et al. 2018).

The functions of the GAR motif and C-terminal BRCT repeats are still elusive as they are not required for DSB repair in the context of BRCA1-deficient cells treated with PARPi, CSR, or dysfunctional telomeres. The 53BP1 BRCT domain can bind to p53 (Derbyshire et al. 2002; Joo et al. 2002) and recent work showed that 53BP1 enhances the p53-dependent transcriptional changes throughout the genome (Cuella-Martin et al. 2016). 53BP1 BRCT repeats also interact directly with γH2AX (Kleiner et al. 2015), but this interaction is not critical for the localization of 53BP1 to sites of DNA damage. However, the C-terminal domain of 53BP1 is important for repair of DSBs in heterochromatin (Noon et al. 2010).

The critical domain with regard to the effects of 53BP1 on DSB repair is the N-terminal region, which contains 28 S/T-Q phosphorylation sites. These sites are phosphorylated by both the ATM and ATR kinases. ATR is activated by RPA coated along ssDNA together with TopBP1 bound to the 9-1-1 clamp at a nearby 5′ ds–ss transition (Saldivar et al. 2017). The ability of 53BP1 to respond to ATR signaling is evident from its localization at telomeres lacking the POT1 component of shelterin, which specifically activate ATR but not ATM signaling (Denchi and de Lange 2007).

As a result of ATM or ATR signaling, the phosphorylated S/T-Q sites in the N terminus of 53BP1 interact with three key effector proteins, each of which requires a distinct set of phosphorylated S/T-Q sites (Fig. 2A). These effectors are (1) Rif1 (ortholog of yeast Rap1-interacting factor 1), which governs processing of DNA ends by recruiting Shieldin, which in turn binds to a complex composed of CST (Ctc1, Stn1, Ten1), Polymerase α, and Primase; (2) Pax2 transactivation domain-interacting protein (PTIP), the function of which remains elusive; and (3) an as-yet-unidentified factor that promotes the mobility of DNA ends. The contribution of these factors varies depending on the context of the DSBs being repaired as discussed in detail below.

## c-NHEJ of telomeres lacking TRF2

**Figure 3.**
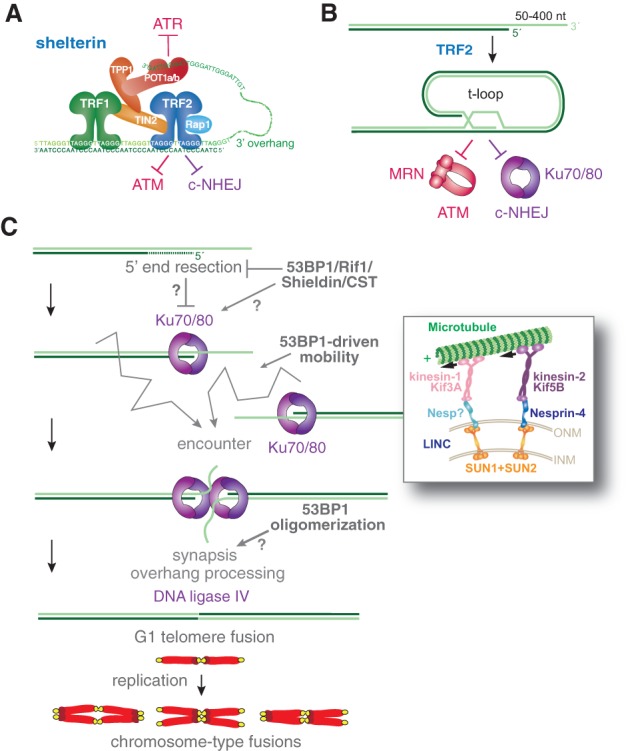
53BP1-dependent c-NHEJ of telomeres lacking TRF2. (*A*) The six-subunit shelterin complex protects telomeres from DNA damage signaling and DSB repair. (*B*) TRF2 in shelterin mediates formation of the protective t-loop by promoting strand invasion of the telomeric 3′ overhang into duplex telomeric DNA. T-loops are proposed to hide the telomere end from the MRN complex and Ku70/80, thereby avoiding initiation of ATM signaling and c-NHEJ. (*C*) Mechanisms by which 53BP1 promotes c-NHEJ-mediated fusion of telomeres lacking TRF2. (*Top*) The Rif1/Shieldin/CST axis promotes telomere fusions. As discussed in the text, the underlying mechanism is unclear. It could involve counteracting 5′ end resection or a more direct mechanism of enhancing c-NHEJ. (*Middle*) 53BP1-driven mobility is proposed to increase the chance that two telomeres (which are distributed throughout the nucleus) can encounter each other and fuse. The *inset* shows the involvement of the LINC complex and cytoplasmic microtubules in the 53BP1-dependent mobility. (*Bottom*) 53BP1-dependent oligomerization and synapsis may further promote the fusion of telomeres that have become closely apposed. Telomere fusions after TRF2 deletion occur predominantly in G1, and after DNA replication are visible as chromosome-type fusions in metaphase spreads.

The joining of dysfunctional telomeres was the first of the three specialized c-NHEJ contexts where a striking dependency on 53BP1 was observed (Dimitrova et al. 2008). Telomeres are protected by the six-subunit shelterin complex, which represses DNA damage signaling, DNA repair, and 5′ end hyperresection (Fig. 3A; de Lange 2018). Shelterin is highly compartmentalized such that different DDR pathways are activated at telomeres depending on which shelterin subunit is removed. When TRF2 is deleted from mouse cells, telomeres activate the ATM kinase signaling cascade in a manner dependent on the MRN complex (Celli and de Lange 2005; Denchi and de Lange 2007; Attwooll et al. 2009; Deng et al. 2009; Dimitrova and de Lange 2009) and the telomeres are joined by Ku70/80- and Lig4- dependent c-NHEJ (Smogorzewska et al. 2002; Celli et al. 2006). TRF2 remodels telomeres into the t-loop and it is thought that the occlusion of the chromosome end in this structure denies MRN and Ku70/80 access to the telomere terminus, thereby preventing activation of ATM and blocking c-NHEJ (Fig. 3B; Griffith et al. 1999; Doksani et al. 2013). Remarkably, absence of 53BP1 (or its upstream regulators ATM, MDC1, and RNF8) (Dimitrova and de Lange, 2006, 2009; Denchi and de Lange 2007; Peuscher and Jacobs 2011) decreases the rate of telomere fusion to the same extent as Lig4 deficiency. This is due to several separable effects of 53BP1: the promotion of chromatin mobility, an effect of the oligomerization domain that may involve telomere clustering, and a somewhat mysterious third function involving Rif1/Shieldin/CST (Figs. 2B, 3C).

Most of the c-NHEJ of telomeres lacking TRF2 occurs in G1, resulting in chromosome-type telomere fusions in metaphase after replication of the fused chromosomes (Fig. 3C). Chromatid-type telomere fusions, which indicate that c-NHEJ took place after DNA replication, are observed but are notably less frequent. In G1, deprotected telomeres behave like one-ended DSBs, analogous to a reversed replication fork in S phase. Since telomeres are distributed throughout the nucleus, telomere–telomere fusion in G1 requires that deprotected telomeres migrate in the nucleus until they encounter a fusion partner.

Telomere–telomere fusion in G1 is stimulated by the ability of 53BP1 to promote the mobility of deprotected telomeres (Fig. 3C). Telomeres that have lost TRF2 become more mobile and roam larger territories than functional telomeres, presumably increasing the chance that they encounter one another (Dimitrova et al. 2008). While the effect of 53BP1 on chromatin mobility was first identified in the setting of deprotected telomeres, 53BP1 also promotes the mobility of IR-induced DSBs (Lottersberger et al. 2015). The underlying mechanism of this dynamic behavior involves a specific set of S/T-Q sites in 53BP1 (Fig. 2A), microtubule dynamics, kinesins, as well as nesprins and the transmembrane SUN1 and SUN2 proteins in the LINC (linker of nucleoskeleton and cytoskeleton) complex (see inset in Fig. 3C; Lottersberger et al. 2015). The S/T-Q interacting partner(s) involved in promoting the mobility of damaged chromatin has not been identified and there is no known protein-protein interaction that links 53BP1 to the LINC complex. The interactions of 53BP1 with PTIP or Rif1 are not required for this function, and neither are the LC8, OD, and BRCT domains (Zimmermann et al. 2013; Lottersberger et al. 2015).

When deprotected telomeres persist for a long time (days), as is the case in c-NHEJ-deficient (*Lig4*^−/−^) cells, a 53BP1-dependent process causes them to become clustered (Timashev et al. 2017). This phenotype may reflect the ability of 53BP1 to hold different sites of DNA damage together (synapsis) in what may be a phase-separated compartment (Kilic et al. 2019). This clustering has also been observed with genome-wide DSBs (Aten et al. 2004). In the context of V(D)J recombination, the synapsis function of 53BP1 is needed for c-NHEJ of RAG-induced DSBs, but only when the DSBs are at a great distance (Difilippantonio et al. 2008). Interestingly, deletion of the OD domain, which has been implicated in synapsis in V(D)J recombination, also diminished the rate of telomere fusions (Fig. 3C; Lottersberger et al. 2013).

The third manner in which 53BP1 promotes telomere fusions involves the Rif1/Shieldin/CST axis (Fig. 3C), pointing to some structural modification or protection of the telomere end. Telomeres, including those lacking TRF2, contain a sizeable 3′ overhang with a minimal length of 50 nt and reaching as long as 400 nt. In mouse cells lacking TRF2, this overhang is removed during c-NHEJ and this processing is strictly dependent on Ku70/80 and Lig4 (Fig. 3C; Celli and de Lange 2005; Celli et al. 2006). The nuclease(s) involved in 3′ overhang removal have remained elusive although MRE11 is a candidate (Deng et al. 2009). Given that overhang processing is coupled to c-NHEJ, the initial presence of this ssDNA is clearly not an impediment to the binding of Ku70/80 to deprotected telomeres.

When TRF2 is inhibited with a dominant-negative (DN) allele that prevents endogenous TRF2 from binding to telomeric DNA, the situation is different. For unknown reasons, the fusions induced by the TRF2-DN allele primarily take place after DNA replication (in S/G2), giving rise to chromatid-type fusions in metaphase (van Steensel et al. 1998). In this setting, overhang removal takes place prior to the initiation of c-NHEJ (van Steensel et al. 1998), suggesting that in G2, Ku70/80 only acts on telomere ends lacking ssDNA (Zhu et al. 2003). The explanation for the inhibitory effect of telomeric overhangs in S/G2 is likely found in CYREN/MRI (Arnoult et al. 2017). This small Ku70-binding protein inhibits the ability of Ku70/80 to act on DSBs with 3′ or 5′ protrusions. If CYREN/MRI only exerts this effect in S/G2, it could explain the cell cycle-dependent effect of the telomeric 3′ overhang on c-NHEJ. The issue is slightly complicated by the fact that CYREN/MRI also acts as a positive regulator of c-NHEJ in G1 (Hung et al. 2018). Nonetheless, there is evidence that in S/G2, the XPF/ERCC1 flap endonuclease is required for the removal of the 3′ overhang prior to the initiation of c-NHEJ (Zhu et al. 2003). This role for XPF in telomere fusions is consistent with CYREN/MRI inhibiting c-NHEJ at DSBs with overhangs in S/G2.

**Figure 4.**
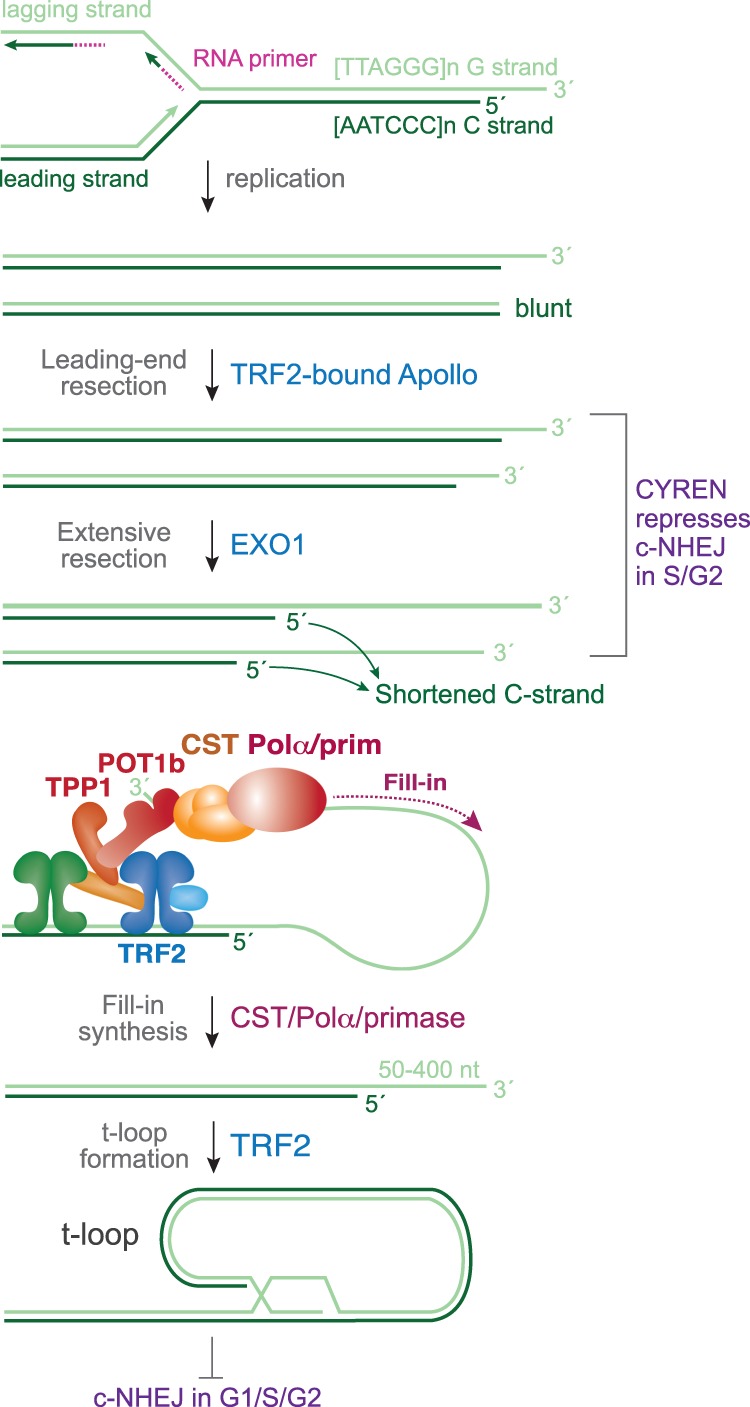
Mechanism of telomere end processing after DNA replication. Telomeres generated by lagging-strand synthesis are assumed to have a short 3′ overhang after RNA primer degradation, while telomeres generated by leading-strand synthesis are initially blunt. Processing by TRF2-bound Apollo results in short 3′ overhangs at leading telomeres, such that both telomere types are substrates for further resection by EXO1. If not counteracted, C-strand attrition due to EXO1 leads to telomere loss. The TPP1/POT1 heterodimer in shelterin recruits the CST/Polα/Primase complex to fill in the resected ends, resulting in a moderately sized (50–400 nt) 3′ overhang that is competent for strand-invasion and t-loop formation.

An important consideration regarding the c-NHEJ of dysfunctional telomeres in G2 is the mechanism of telomere end processing after DNA replication (Fig. 4). As telomeres require a 3′ overhang for t-loop formation and thus for their protection, this structure needs to be regenerated at both sister telomeres. The sister telomere generated by leading-strand DNA synthesis (referred to as the leading-end telomere), is first processed by Apollo, a TRF2-bound nuclease, to yield a short overhang that may be akin to the product of MRN/CtIP at DSBs (Lam et al. 2010; Wu et al. 2010). Next, both sister telomeres are extensively resected by EXO1, resulting in extended overhangs (Wu et al. 2012). If not counteracted, this hyperresection of the 5′ ends can lead to truncated telomeres (Surovtseva et al. 2009; Price et al. 2010; Takai et al. 2016). Such telomere loss is avoided by a final step where the POT1/TPP1 components of shelterin recruit CST/Polα/Primase to mediate fill in of hyperresected ends (Wu et al. 2012). This process creates telomeres with moderately sized 3′ overhangs that can form t-loops.

When TRF2 is deleted from telomeres in cells lacking Rif1, the telomeric overhangs become twofold longer and the rate of telomere fusions is diminished by approximately twofold (Zimmermann et al. 2013). A similar effect is observed when Shieldin or CST is depleted (Boersma et al. 2015; Xu et al. 2015; Barazas et al. 2018; Dev et al. 2018; Gupta et al. 2018; Mirman et al. 2018; Noordermeer et al. 2018). One interpretation of this result is that the longer overhangs are an impediment to Ku70/80 loading (Fig. 3C). However, most telomere fusions studied in these experiments take place in G1 when Ku70/80 is not repressed by CYREN/MRI. Why would a doubling of the overhang length (i.e., from 50–400 nt to 100–800 nt) inhibit c-NHEJ? Perhaps Ku70/80 is particularly sensitive to a doubling of the overhang length, or perhaps the longer overhangs form secondary structures (e.g., G4 structures) and/or bind proteins that impede c-NHEJ. Another possibility is that it is not the length of the overhang that affects the repair outcome but rather the presence of the Shieldin/CST/Polα/Primase complex on the telomeric overhang. Perhaps the Shieldin/CST/Polα/Primase complex blocks proteins (e.g., Rad51 or Rad52) that can compete with c-NHEJ factors. The presence of CST at telomeres is known to block telomerase (Chen et al. 2012; Hockemeyer and Collins 2015), so this type of inhibitory effect is not unprecedented. Further work on the effect of long overhangs at dysfunctional telomeres and other DSBs on c-NHEJ in G1 is needed to resolve these issues.

## c-NHEJ of AID-induced DSBs in class switch recombination

**Figure 5.**
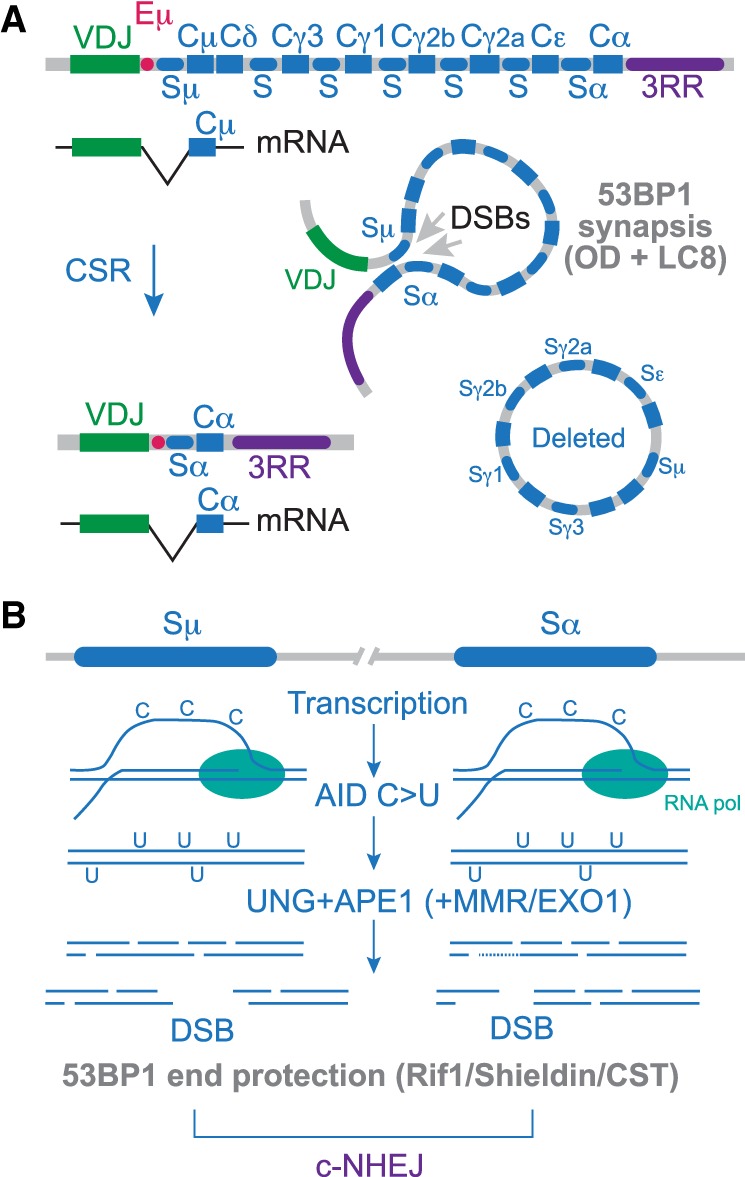
The role of 53BP1 in class switch recombination (CSR). (*A*) Schematic of immunoglobulin locus. Rectangles represent exons and ovals represent switch regions in the introns. In this example, AID creates DSBs in Sμ and Sα leading to a switch of Cμ to Cα. The synapsis function of 53BP1 contributes to the proximity of the DSBs. (*B*) Mechanism of DSB formation in CSR. Transcription of the switch regions creates the ssDNA substrate for AID-mediated cytosine deamination. The resulting uracil residues are processed by base excision repair (BER) and mismatch repair (MMR) resulting in frequent nicks that can lead to DSB formation. These DSBs can carry 5′ or 3′ overhangs and require the Rif1/Shieldin/CST axis of 53BP1 for efficient joining. As is the case for telomere fusions, the exact role of Rif1/Shieldin/CST is not clear (see the text).

CSR is a programmed recombination in the immunoglobulin locus that allows B cells to switch between different classes of antibodies (Fig. 5A; Methot and Di Noia 2017). This remarkable process involves Ku70/80-, DNAPKcs-, Lig4-, and XRCC4/XLF-dependent c-NHEJ repair of DSBs generated in the switch regions. Switch regions are positioned in the introns preceding exons that encode the various classes of immunoglobulin heavy chains. Transcription of the highly repetitive, GC-rich switch regions is required for CSR. In CSR, the repair of DSBs by c-NHEJ is unusual in that they are far apart (20–600 kb). For CSR to occur, DSB repair needs to create deletions, rather than nonproductive intraswitch DSB repair (or inversions) (Fig. 5A).

The DSBs required for CSR are generated by activation-induced deaminase (AID), a member of the family of APOBEC cytosine deaminases (Fig. 5B). AID deaminates cytosine in ssDNA to uracil, which is converted to a strand break after uracil removal by uracil DNA glycosylase (UNG) and cleavage of the resulting abasic site by the base excision repair nuclease APE1. Cytosine deamination takes place on both the template and nontemplate strands (Basu et al. 2011) so that closely positioned nicks on opposite strands can result in DSBs. In addition, recognition of the U:G mismatch by the mismatch repair (MMR) MSH2/6 complex and subsequent exonucleolytic attack by EXO1 on nicks on the opposing strand (perhaps generated by APE1) appears to enhance DSB formation in CSR (Bregenhorn et al. 2016; Methot and Di Noia 2017). The DSBs formed through this process are predicted to contain either 5′ or 3′ overhangs of variable lengths (Fig. 5B). As CSR takes place in G1 (Petersen et al. 2001) when CYREN/MRI is not inhibitory to c-NHEJ (Arnoult et al. 2017), Ku70/80 should be able to act on DSBs despite the presence of 5′ and 3′ overhangs. Some overhang processing will be needed during c-NHEJ, but the nucleases involved are not yet known.

CSR has been a great tool for dissecting the functions of 53BP1. In the absence of 53BP1, CSR is severely impaired and the recombination in the Ig locus shifts to intraswitch repair events that are accompanied by extensive resection (Franco et al. 2006; Reina-San-Martin et al. 2007; Bothmer et al. 2010). Two distinct aspects of 53BP1 promote CSR (Figs. 2B, 5). First, CSR requires the ability of 53BP1 to form stable oligomeric assemblies at sites of DNA damage. This attribute is dependent on the cooperation of the two LC8 motifs with the OD (Bothmer et al. 2011; Lottersberger et al. 2013; Becker et al. 2018; Sundaravinayagam et al. 2019). The LC8 motifs, through their interaction with DYNLL1, promote oligomerization in vitro and enhance the stability of 53BP1 at sites of DNA damage. The LC8 motifs alone are insufficient and CSR requires the simultaneous oligomerization of 53BP1 through its OD (Becker et al. 2018). The most likely explanation for CSR dependence on higher order 53BP1 assemblies is that distal DSBs need to be held in proximity for a productive c-NHEJ event (Fig. 5A).

The second main determinant of 53BP1-promoted CSR is found in Rif1/Shieldin/CST (Fig. 5B). Loss of any one of these components or failure of 53BP1 to bring these factors to DSBs reduces CSR (Chapman et al. 2013; Di Virgilio et al. 2013; Escribano-Diaz et al. 2013; Boersma et al. 2015; Xu et al. 2015; Barazas et al. 2018; Dev et al. 2018; Ghezraoui et al. 2018; Gupta et al. 2018; Noordermeer et al. 2018). Molecular analysis of the few CSR products formed in the absence of Rif1/Shieldin/CST shows that there is extensive resection (Chapman et al. 2013; Di Virgilio et al. 2013; Ghezraoui et al. 2018). The requirement for Rif1/Shieldin/CST in CSR is paradoxical since CSR takes place at DSBs that already carry various ssDNA protrusions. Why then, are proteins involved in minimizing 3′ overhang length needed? This question is analogous to that posed above in the context of telomere fusions where Rif1/Shieldin/CST promote c-NHEJ despite the fact that 3′ overhangs do not inhibit c-NHEJ. Of particular interest is the observation that the Shieldin complex coevolved with CSR (Gupta et al. 2018). This suggests that Shieldin is a relatively recent elaboration of the 53BP1 repertoire that has evolved to facilitate CSR.

## c-NHEJ of DSBs in PARPi-treated BRCA1-deficient cells

The context of 53BP1 action that has strongly influenced current models of 53BP1 function is that of BRCA1-deficient cells treated with PARP1 inhibitors. BRCA1 plays a critical role in HDR at multiple steps (Wright et al. 2018). BRCA1 is proposed to facilitate 5′ end resection and promotes the loading of Rad51 by BRCA2. Together with a role in protecting replication forks (Ray Chaudhuri et al. 2016), the requirement for BRCA1 in HDR is thought to underlie the etiology of BRCA1-deficient cancers. Seminal work revealed that cells deficient in BRCA1 are highly sensitive to PARPi (Bryant et al. 2005; Farmer et al. 2005) and this synthetic lethality is now being exploited clinically (Fong et al. 2009; Tutt et al. 2010; Pilié et al. 2019).

**Figure 6.**
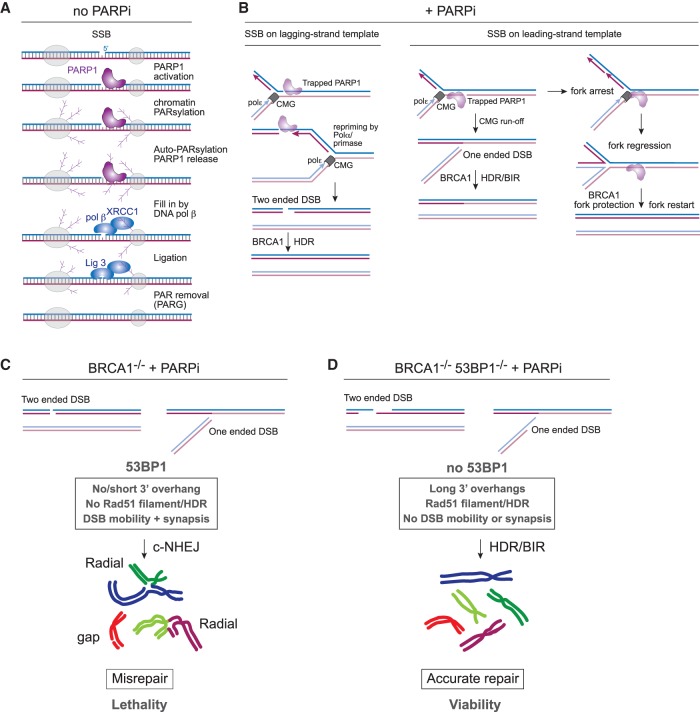
Mechanism of action for PARPi and synthetic lethal genetic interaction with BRCA1. (*A*) The role of PARP1 in recognition and repair of SSBs. (*B*) The effects of PARPi on SSBs on lagging- or leading-strand template during DNA replication. “PARP-trapping” occurs when PARP1 remains bound to the SSB due to inhibition of its autoPARsylation activity by PARPi. (*C*) The effects of simultaneous disruption of BRCA1 and PARP1. In the absence of BRCA1-mediated HDR, 53BP1 mediates misrepair of broken chromosomes, leading to cellular death. (*D*) Concurrent loss of 53BP1 restores HDR, leading to resistance to PARPi in BRCA1-deficient cells.

PARP1 is required for the repair of single-stranded breaks (SSBs) that result from oxidative damage and are formed during base excision repair (BER) (Barnes and Lindahl 2004; Ray Chaudhuri and Nussenzweig 2017), a pathway that removes thousands of aberrant nucleotides per genome each day (Fig. 6A). PARP1 binds to and is activated by SSBs. Once active, PARP1 will promiscuously PARsylate chromatin constituents resulting in a local network of branched PAR chains. PAR functions to recruit XRCC1, polymerase β, and ligase 3, which repair the SSB. PARP1 also PARsylates itself, allowing the enzyme to evacuate the lesion. When PARP1 activity is inhibited, PARP1 can become locally trapped at the break, preventing further repair (Pommier et al. 2016; Zimmermann et al. 2018). The resulting lesions, with or without trapped PARP1, generate DSBs and/or impede fork progression during DNA replication, but the trapping of PARP1 is thought to be the major source of the effect of PARPi on BRCA1-deficient cells (Fig. 6B).

When a replication fork encounters an SSB, several outcomes are possible (Fig. 6B). If the lesion is on the lagging-strand template, a two-ended DSB may result after repriming by Polα (Taylor and Yeeles 2018). If the leading-strand template has an SSB, no bypass is possible and a one-ended DSB will be formed. Both types of DSBs require HDR for precise repair and therefore depend on BRCA1. In BRCA1-deficient cells, some of the two-ended DSBs may be repaired by c-NHEJ without impairing cell viability. However, c-NHEJ of one-ended DSBs can give rise to aberrant repair products as can misrejoining of two-ended DSBs. Specifically, if a DSB (one- or two-ended) becomes ligated to a DSB on another chromosome, a lethal type of chromosome aberration is formed (Fig. 6C). These aberrant joining events are evident in metaphase chromosome spreads where they are visible as multi-armed misrejoined chromosomes, often referred to as “radial chromosomes.” (Note: “radial chromosomes” has become an accepted term to refer to triradial and quadriradial chromosomes that have chromosome arms extending into three or four rather than the regular two opposing directions [see, for instance, Oostra et al. 2012].) Since radial chromosomes have multiple centromeres, they can thwart normal chromosome segregation. Cells harboring radial chromosomes attempt to undergo cell division but succumb to some form of mitotic catastrophe and show hallmarks of mitotic dysfunction including anaphase bridges and micronuclei (Schoonen et al. 2017). Lack of proper repair of the PARPi-induced DSBs is also evident from gaps and breaks in metaphase chromosomes (Fig. 6C).

As is the case with dysfunctional telomeres and CSR, several aspects of 53BP1 conspire to promote PARPi sensitivity of BRCA1-deficient cells (Figs. 2B, 6). First, formation of radial chromosomes requires that a DSB on one chromosome reaches a DSB on another. It is therefore not surprising that the ability of 53BP1 to promote the mobility of DSBs contributes to the synthetic lethality of PARPi with BRCA1 deficiency (Lottersberger et al. 2015), as does the synapsis function of 53BP1 (Bothmer et al. 2011). The second mechanism by which 53BP1 promotes PARPi sensitivity involves the Rif1/Shieldin/CST/Polα/Primase axis. When this function of 53BP1 is disabled, BRCA1-deficient cells regain their ability to repair PARPi-induced DSBs by HDR and survive (Chapman et al. 2013; Escribano-Diaz et al. 2013; Feng et al. 2013; Zimmermann et al. 2013; Xu et al. 2015; Barazas et al. 2018; Dev et al. 2018; Gao et al. 2018; Ghezraoui et al. 2018; Gupta et al. 2018; Mirman et al. 2018; Noordermeer et al. 2018). The proposed mechanism for this reactivation of HDR is discussed below.

A factor whose contribution remains unclear is PTIP. Deletion of PTIP rescues the formation of PARPi-induced radials and lethality in BRCA1-deficient cell lines (Callen et al. 2013; Wang et al. 2014; Ray Chaudhuri et al. 2016). This presents a conundrum since 53BP1 clearly also mediates the effects of PARPi through promoting DSB mobility and synapsis as well as through Rif1/Shieldin/CST/Polα. There is no crosstalk between PTIP and either Rif1 or the induction of DSB mobility that can explain the discrepancy. In addition, some of the effects of PTIP in BRCA1-deficient cells do not appear to require its interaction with 53BP1 (Ray Chaudhuri et al. 2016). Indeed, an allele of 53BP1 that does not bind PTIP continues to promote radial formation and lethality in PARPi-treated BRCA1-deficient cells unless the Rif1/Shieldin axis is also absent (Callen et al. 2019). Perhaps once the mechanism by which PTIP promotes PARPi-induced radials and lethality is known, it will become clear why deletion of PTIP has the same effect as deletion of 53BP1.

In addition to formation of DSBs, trapping of PARP1 at SSBs could create a barrier to replication that results in fork arrest (Fig. 6B). The contribution (if any) of these PARPi-induced fork-stalling events to the formation of radials and accompanying lethality is not known. However, it is noteworthy that in hydroxyurea (HU)-treated cells, the stability of arrested forks requires BRCA1 (Schlacher et al. 2011, 2012; Ray Chaudhuri et al. 2016; Taglialatela et al. 2017; Rickman and Smogorzewska 2019). In the absence of BRCA1, a small percentage of HU-induced fork arrests are processed into chromosomal aberrations. BRCA1 blocks degradation of arrested forks by MRE11 (Schlacher et al. 2012), which is recruited there by PTIP (Ray Chaudhuri et al. 2016). However, in this context PTIP acts independently of 53BP1, and 53BP1 itself has no effect of the outcome of HU treatment in BRCA1-deficient cells (Ray Chaudhuri et al. 2016). Therefore, it remains unclear how the events induced by HU relate to those induced by PARPi.

An additional point of consideration is the mutually antagonistic relationship between BRCA1 and 53BP1 that affects each factor's accumulation near DSBs. Independently of BRCA1, the presence of 53BP1 at DSBs is down-modulated in S phase such that there are fewer and/or less extensive sites of 53BP1 accumulation in newly replicated chromatin where the H4K20Me marks are diluted (Saredi et al. 2016; Nakamura et al. 2019). In G2, 53BP1 foci regain their prominence (Simonetta et al. 2018). In addition, BRCA1 changes the nature of 53BP1 foci in S phase, converting them from homogeneous domains into more hollow spheres, suggesting that BRCA1 has the ability to relegate 53BP1 to the outskirts of the DDR-marked chromatin (Chapman et al. 2012; Escribano-Diaz et al. 2013). Conversely, 53BP1 appears to minimize the accumulation of BRCA1 at DSBs in G1 in a manner that involves Rif1 and the Rev7 component of Shieldin (Escribano-Diaz et al. 2013; Feng et al. 2013; Zimmermann et al. 2013; Simonetta et al. 2018). Recent high-resolution imaging of DNA damage foci reveals a complex architecture with different domains occupied by BRCA1, 53BP1, and Rif1 (Ochs et al. 2019). Interestingly, 53BP1 and Rif1, but not Shieldin, are involved in stabilizing damage-proximal chromatin into globular subdomains within DNA damage foci (Ochs et al. 2019). The mechanism of the mutual antagonism between 53BP1 and BRCA1 may emerge from further dissection of this interplay.

## Two models for the role of the Rif1 axis

**Figure 7.**
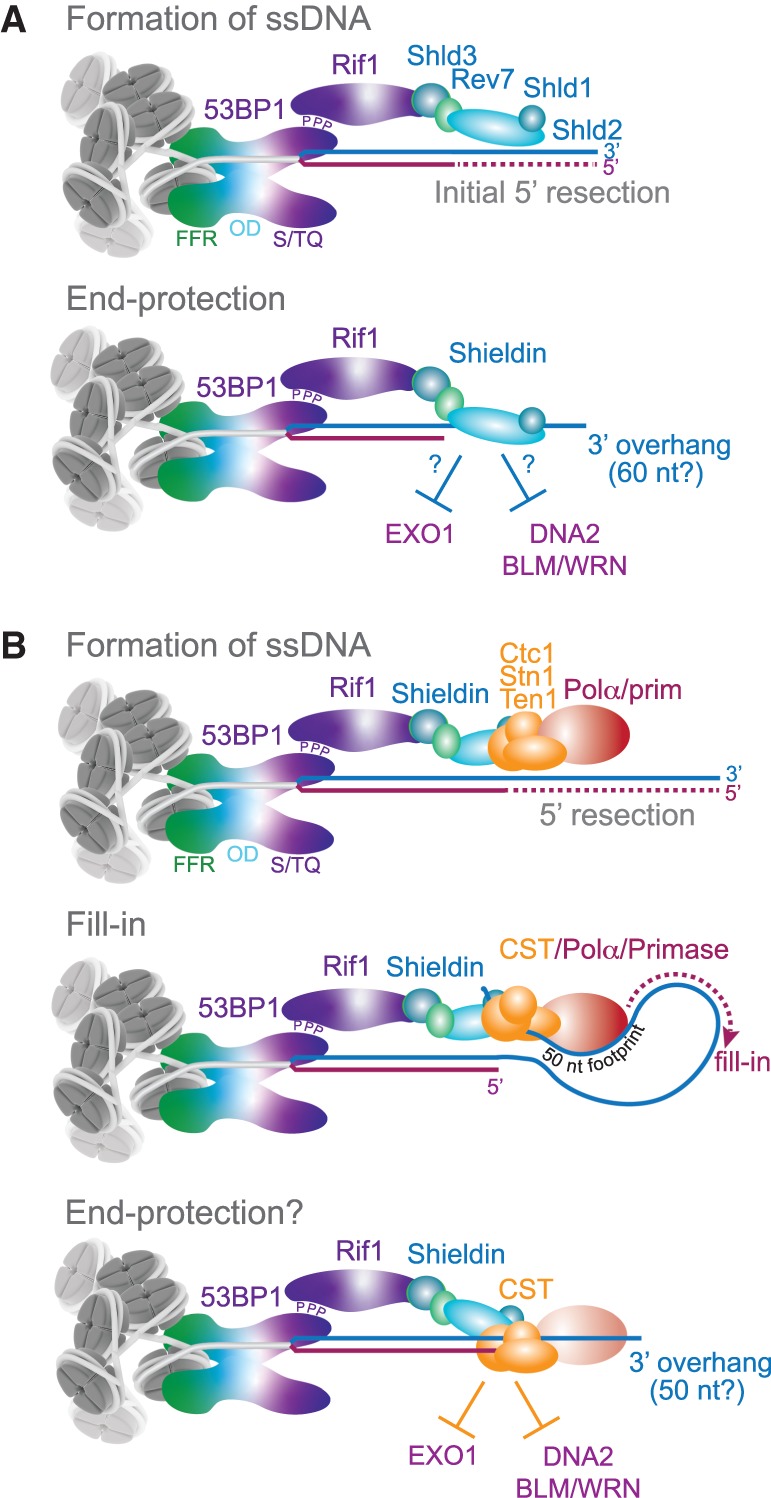
Two models for the role of the Rif1 axis. (*A*) End protection by the ssDNA-binding Shieldin complex limits resection by EXO1 and BLM/WRN/DNA2. (*B*) Shieldin recruits CST/Polα/Primase, executing a fill-in reaction to counteract resection. This model is also compatible with an additional step of end protection by Shieldin/CST after fill in. Note that in both models, initial 5′ end resection occurs to allow ssDNA binding by Shieldin and CST, and a moderately sized (∼50 nt) 3′ overhang is predicted to remain.

Concerning the mechanism by which 53BP1 limits the formation of ssDNA at DNA breaks, there are two main models (Fig. 7; Barazas et al. 2018; Dev et al. 2018; Findlay et al. 2018; Gao et al. 2018; Ghezraoui et al. 2018; Gupta et al. 2018; Mirman et al. 2018; Noordermeer et al. 2018). In the first model, 53BP1 uses the loading of Shieldin onto the ssDNA to protect the 5′ end from resection (Fig. 7A). The finding that Shld2 alone or in complex with Shld1 can bind to ssDNA is promising in this regard (Dev et al. 2018; Findlay et al. 2018; Gao et al. 2018; Noordermeer et al. 2018), but it remains to be seen whether Shieldin binds sufficiently close to the 5′ end to form a barrier to nucleolytic attack. For Shieldin to block resection upon engaging the DNA end, at least some ssDNA must already be present. The length of this 3′ overhang is not yet clear since the minimal binding site of the whole Shieldin complex has not been determined. In vitro, Shld2/Shld1 complexes bind to oligonucleotides of 60–100 nt (Dev et al. 2018; Findlay et al. 2018; Gao et al. 2018; Noordermeer et al. 2018).

Assuming there is sufficient ssDNA for Shieldin to bind at the DSB, how would it prevent additional resection? Perhaps Shieldin simply hides the 5′ end from nucleases. A second possibility is that Shieldin terminates EXO1 resection in the same way that loading of RPA on 3′ overhangs inhibits further resection by EXO1 (Nicolette et al. 2010; Yang et al. 2013; Myler et al. 2016; Gong et al. 2017; Soniat et al. 2019). In addition, resection by DNA2 in conjunction with either WRN or BLM could be inhibited if Shieldin prevents the loading of RPA, which normally stimulates these RecQ helicases (Nimonkar et al. 2011). Even though Shieldin is much less abundant than RPA and has a lower affinity for ssDNA, it might effectively outcompete RPA due to its tethering to 53BP1. The same principle of local tethering allows POT1, the ssDNA-binding protein in shelterin, to outcompete the more abundant RPA (Kratz and de Lange 2018).

In the second (not mutually exclusive) model, Shieldin functions to recruit CST/Polα/Primase (Fig. 7B). The recruitment of CST/Polα/Primase could affect the structure of the DNA in two ways. First, CST is known to bind with high affinity to ds–ss junctions (Bhattacharjee et al. 2017), potentially allowing the complex to protect 5′ ends from EXO1 and block access of the BLM and WRN helicases. As is the case with Shieldin, CST binding at a DSB will require a 3′ overhang (for CST, in the range of 10–18 nt) (Bhattacharjee et al. 2017). CST tends to favor sequences with runs of G residues (Hom and Wuttke 2017), which would restrict its binding opportunities in short 3′ overhangs. It is also possible that the CST binding specificity is altered when it is bound to Shieldin, allowing it to engage overhangs without G runs.

In addition to CST simply protecting the 5′ end from resection activities, CST can counteract resection (Fig. 7B). CST is known to promote Polα and Primase activity (Goulian et al. 1990; Casteel et al. 2009; Ganduri and Lue 2017), allowing fill-in synthesis to occur. This fill-in is relevant to the outcome of PARPi-treatment of BRCA1-deficient cells. Inhibition of Polα or Primase leads to a reduction in radials formed after PARPi treatment (Mirman et al. 2018; Z Mirman and T de Lange, in prep.). Furthermore, BrdU incorporation can be detected at DSBs and this DNA synthesis is dependent on 53BP1, Shieldin, CST, Polα, and Primase (Z Mirman and T de Lange, in prep.). Polα has limited processivity and usually synthesizes ∼20–25 nt (Pellegrini 2012). During canonical DNA replication, these products are extended by Polδ, but whether Polδ is involved at DSBs is unknown. Perhaps repeated fill-in steps by Polα can convert long overhangs into dsDNA.

The CST/Polα/Primase fill-in reaction is predicted to leave a considerable 3′ overhang because of the removal of the RNA primer and the inability of Polα to copy the region where CST (and Shieldin) is bound. The residual overhang left by this “footprint” may be as long as 50 nt. At telomeres, where CST plays an analogous role in counteracting resection, the fill-in reaction indeed allows retention of overhangs of at least 50 nt (Makarov et al. 1997; Wright et al. 1997; Sfeir et al. 2005; Wu et al. 2012). If CST also has the ability to protect 5′ ends from further resection, the outcome would be DNA ends with overhangs of up to 50 nt but not much longer (Fig. 7B).

Regardless of the mechanism by which the Rif1/Shieldin/CST axis acts, a DSB that is acted upon by 53BP1 is predicted to carry a 3′ overhang. According to these considerations, 53BP1 does not actually prevent the formation of 3′ overhangs. Rather, 53BP1 appears to prevent the formation of 3′ overhangs that are “overly long” (>50 nt in the CST model; perhaps as long as 60 nt in the Shieldin model).

## The 53BP1 dilemma

The selective advantage of 53BP1 is an enigma (at least to these authors) because most of its described functions are not obviously beneficial. 53BP1 cannot have evolved solely to promote CSR since it was present long before the innovation of CSR in the adaptive immune system (Gupta et al. 2018). Obviously, 53BP1 also did not evolve to mediate telomere fusions or make BRCA1-deficient cells sensitive to PARPi. Furthermore, several of 53BP1's attributes are potentially disastrous for cells with multiple DSBs. The propensity of 53BP1 to hold together and/or cluster DSBs that are at a distance is expected to promote translocations with potential cancer-causing consequences. Similarly, promoting the mobility of DSBs could be expected to engender misrepair.

What is the purpose of 53BP1's ability to limit the extent of ssDNA at DSBs? It has been argued (including by one of us) (Zimmermann and de Lange 2014) that this function of 53BP1 is needed for the control of DSB repair, directing DSBs away from HDR and toward c-NHEJ in G1. However, if 53BP1 limits resection by loading Shieldin (with or without CST) on the ssDNA, the ssDNA occupied by Shieldin/CST would be removed during the subsequent c-NHEJ. It seems difficult to argue that 53BP1 has evolved to promote a form of c-NHEJ that is always accompanied by deletions. Such a system would only make sense in the context of CSR where small deletions are not detrimental. Furthermore, the danger of HDR in G1, and hence the need for 53BP1 to shuttle DSBs into c-NHEJ, is limited. For instance, BRCA2 does not accumulate at sites of DNA damage before entry into S phase, even in the absence of 53BP1 (Orthwein et al. 2015), and initiation of resection by MRN/CtIP is under cell cycle control (Sartori et al. 2007; Huertas and Jackson 2009).

In summary, 53BP1 prevents formation of long 3′ overhangs at DSBs and alters DSB dynamics in a manner that could promote translocations. Below, we argue that these potentially detrimental attributes of 53BP1 are best understood as a mechanism to ensure the fidelity of DSB repair rather than a mechanism to channel DSBs into c-NHEJ at the expense of HDR.

## Speculation: 53BP1 as a DSB escort that promotes repair fidelity and CSR

The considerations above led us to speculate that the selective advantage of mammalian 53BP1 resides in its ability to block illegitimate recombination. We further speculate that several of the current-day attributes of 53BP1 coevolved with CSR.

**Figure 8.**
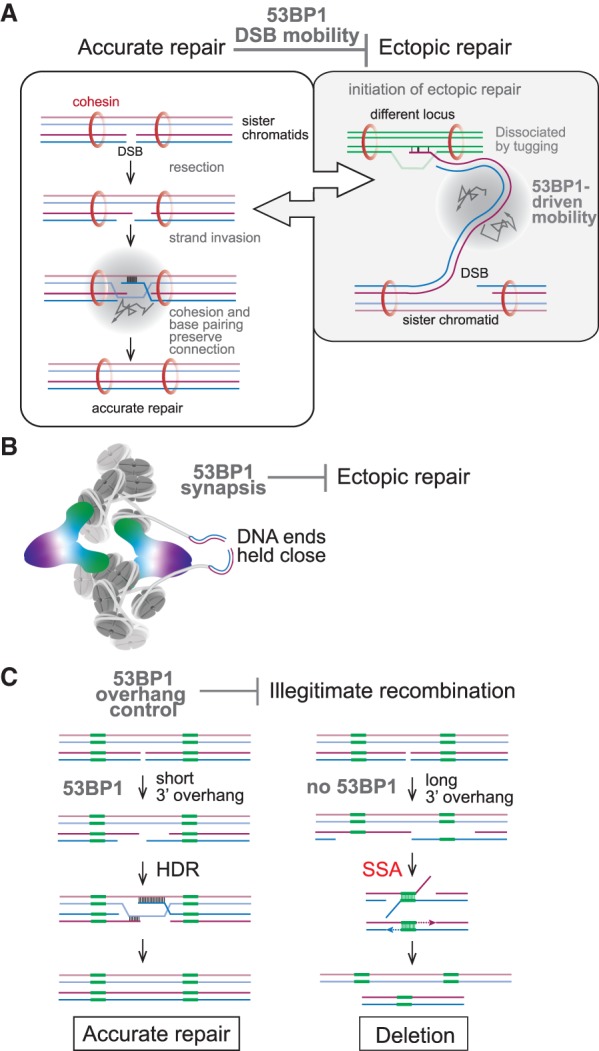
Model for 53BP1 as a DSB escort that promotes repair fidelity and CSR. (*A*) 53BP1-driven mobility of chromatin containing a DSB is proposed to discourage ectopic repair. (*Left*) S-phase DSBs are preferentially repaired by HDR using the sister chromatid. (*Right*) If a DSB becomes disconnected from the sister chromatid (perhaps due to movements created by transcription or loop extrusion), ectopic repair could be initiated. 53BP1-driven chromatin mobility is proposed to dislodge such ectopic interactions, allowing the DSB to rejoin the sister chromatid. When the DSB is engaged in repair using the sister chromatid, 53BP1-driven mobility will not dislodge the DSB because of the counterforce of cohesion and extensive base pairing. (*B*) 53BP1 is proposed to prevent ectopic repair by holding the two DNA ends of a DSB in proximity. (*C*) The Rif1 axis of 53BP1 is proposed to repress illegitimate recombination through SSA by preventing the formation of long 3′ overhangs. Repeats that could lead to SSA (green boxes) are only exposed when 3′ overhangs become overly long in the absence of 53BP1.

The first aspect of 53BP1 that can improve the fidelity of DNA repair is its ability to mobilize DSBs. We have previously argued (Lottersberger et al. 2015) that DSB mobility is a mechanism to counteract ectopic recombination of DSBs (Fig. 8A). In this view, 53BP1 could disengage DSBs that have lost their proximity to the sister chromatid and are invading homologous (e.g., repetitive) sequences at another locus. Processes such as cohesin-mediated loop extrusion and torsional stress induced by transcription could be the source of disengaged DSBs that would need 53BP1-driven mobility to rejoin their original partner. DSBs that are engaged in recombination with the sister chromatid would be resistant to this force because they are held down by cohesion and because of Rad51-mediated strand invasion. DSB mobility in G1 may also allow disconnected DNA ends to undertake a search to reconnect and avoid repair at ectopic sites. DSB mobility could be disadvantageous in cells with a high burden of DNA breaks (such as after IR in laboratory settings or upon removal of TRF2), but in vivo, most nuclei will contain only one or a few DSBs.

A second aspect of 53BP1 that may promote the fidelity of DSB repair is its synapsis function (Fig 8B). By keeping DSBs together until their successful repair, 53BP1 could diminish the risk of ectopic repair in the setting of DSBs in S/G2 and in G1. This function of 53BP1 is also suited for CSR where distal DSBs need to be held together. Therefore, the synapsis function of 53BP1 may have been enhanced when CSR evolved. As is the case for DSB mobility, synapsis can have detrimental side effects when multiple DSBs become clustered, but (as argued above) this situation is unlikely to occur in vivo.

The third aspect of 53BP1 that improves the fidelity of DNA repair is found in the Rif1/Shieldin/CST axis. By limiting the formation of long 3′ overhangs at DSBs, 53BP1 can prevent DSBs from being processed by SSA (Figs. 1B, 8C). SSA is a dangerous repair pathway in genomes with many repetitive sequences, since deletions will be a frequent outcome. Indeed, a shift from HDR to SSA has been noted under conditions where 53BP1 function is insufficient (Escribano-Diaz et al. 2013; Ochs et al. 2016).

Although the Rif1/Shieldin/CST axis can be rationalized based on the proposal that this system has evolved to repress SSA at DSBs, a puzzle remains. As noted above, both in the settings of CSR and telomere fusions, the ability of the Rif1/Shieldin/CST axis to promote c-NHEJ is not readily explained simply based on limiting the length of 3′ overhangs. In addition, the c-NHEJ-mediated formation of radial chromosomes in PARPi-treated BRCA2-null cells that lack 53BP1 argues that in this context too, long 3′ overhangs do not impede c-NHEJ. Is it possible that Rif1/Shieldin/CST have a feature that improves c-NHEJ independent of their effects on ssDNA formation? Perhaps their presence competes with other factors that would shuttle the ends into alternative pathways? For instance, do they compete with Rad51 and/or Rad52, as suggested by Lukas (Ochs et al. 2016)?

If Rif1/Shieldin/CST prevent the binding of other DNA repair factors, this could explain why loss of 53BP1/Rif1/Shieldin/CST promotes HDR in cells lacking BRCA1. Reactivation of HDR is not fully explained based on the creation of ssDNA since it is predicted that the DSBs have an overhang even when Rif1/Shieldin/CST are engaged. It is not excluded that the reactivation of HDR results from both the creation of 3′ overhangs as well as the absence of the Rif1/Shieldin/CST complex at the DNA end. The observation that there are more RPA and Rad51 foci at DSBs in BRCA1-deficient cells when Rif1/Shieldin/CST are absent is compatible with the formation of longer regions of ssDNA, but it is also fully compatible with the idea that Shieldin/CST compete with Rad51 and other ssDNA-binding proteins.

Taking this line of reasoning to its logical conclusion, one function of BRCA1 may be to prevent the persistence of Rif1/Shieldin/CST at DSBs in newly replicated regions where HDR occurs. This view fits with the ability of BRCA1 (in collaboration with CtIP) to minimize 53BP1 at DSBs (Chapman et al. 2012; Escribano-Diaz et al. 2013) and with the diminished recruitment of 53BP1 at DSBs where replication has reduced the abundance of H4K20Me2 (Pellegrino et al. 2017). It is even possible that the purported resection function of BRCA1, which is measured based on ssDNA proxies such as RPA and Rad51, actually reflects its efforts to remove 53BP1 from DSBs thereby allowing HDR to take over.

These speculations suggest testable hypotheses. First, it will be important to test the view that the association of Ku70/80 with DSBs (and thus c-NHEJ) is impeded by 3′ overhangs, a contention that does not appear to fit with the current data on telomere fusions and CSR. Second, it is critical to test the hypothesis that HDR can be repressed if 53BP1 and its downstream ssDNA-binding factors persist at DSBs. Interestingly, after submission of this review, a new report revealed data compatible with the proposed ability of 53BP1 to repress HDR independent of its effect on resection (Callen et al. 2019). Third, the view that 53BP1 primarily acts to improve DSB repair fidelity, for instance by preventing translocations and repressing SSA, can be tested by analyzing the genomic consequences of 53BP1 deficiency in contexts other than the three specialized settings that are discussed above.
